# Responsible Leadership Fuels Innovative Behavior: The Mediating Roles of Socially Responsible Human Resource Management and Organizational Pride

**DOI:** 10.3389/fpsyg.2021.787833

**Published:** 2021-12-10

**Authors:** Wenli Dong, Lifeng Zhong

**Affiliations:** School of Business, Renmin University of China, Beijing, China

**Keywords:** responsible leadership, innovative behavior, socially responsible HRM, organizational pride, sequential mediation

## Abstract

Leaders are under increasing pressure to inspire innovative endeavors in responsible ways. However, whether and how responsible leadership can fuel employee innovative behavior remains unknown. Therefore, drawing on social identity theory and social exchange theory, this study aims to investigate the psychological mechanisms underlying the responsible leadership-innovative behavior relationship. Multi-phase data were collected from 280 employees working in Chinese manufacturing firms to test the hypotheses using hierarchical regression analyses and the bootstrap method. The results reveal that responsible leadership is positively related to innovative behavior. Additionally, perceived socially responsible human resource management (HRM) and organizational pride separately and sequentially mediate the responsible leadership-innovative behavior relationship. This study empirically reveals the effectiveness of responsible leadership and sheds new light on the psychological processes through which it facilitates innovative behavior, revealing the generalizability of responsible leadership and innovative behavior in the Chinese context. Moreover, we respond to the call for incorporating leadership theory into HRM research and further advance the existing knowledge on both antecedents and outcomes of socially responsible HRM. For practical guidance, organizations are encouraged to foster innovation through investment in responsible management practices. Research limitations and implications are also discussed.

## Introduction

Responsibility is one of the key elements for leadership effectiveness in the field of organizational study (Waldman and Galvin, 2008). In today’s interconnected business environment, the absence of leaders’ responsibility has led firms to the crisis of organizational legitimacy and public trust (e.g., Volkswagen emissions scandal) (Maak and Pless, 2006). Therefore, leaders ought to behave more responsibly toward both internal and external stakeholders (e.g., employees, customers, suppliers, communities, and the environment) to achieve long-term success (Maak, 2007). As an ethical and social-relational phenomenon that reaches beyond the traditional leader-subordinate dyadic relationship (Maak and Pless, 2006), responsible leadership is perceived as more effective and can influence organizations more than other leadership styles (Haque et al., 2019b; Voegtlin et al., 2020). However, previous research has mostly concentrated on its pro-social outcomes (Voegtlin et al., 2020; Ullah et al., 2021; Zhang et al., 2021). Few studies have empirically investigated how responsible leadership affects employees’ work-related behavior (Haque et al., 2019b), especially innovative behavior that contributes much to organizational innovation and competitive advantage (Scott and Bruce, 1994). Hence, this study endeavors to bridge the gap by focusing on how responsible leadership influences employee innovative behavior in a sample of Chinese manufacturing firms. In China, the manufacturing industry occupies a vital position in the national economy (about 30% of GDP) (Feng et al., 2018). The rapid development of industrial modernization since the 1980s requires manufacturing managers to act more responsibly toward various stakeholders to balance economic, environmental, and social performance (Zhu and Sarkis, 2004). Since 2019, manufacturing firms have accounted for 41.25% of China’s top 500 list of corporate social responsibility (CSR). Furthermore, in the context of industry 4.0 dominated by intelligent manufacturing (Feng et al., 2018), manufacturing firms in China are under increasing pressure to achieve sustainable innovation (Wang et al., 2021). Hence, exploring the responsible leadership-employee innovation linkage plays an important role in addressing such challenges, and this study aims to reveal whether and how responsible leadership affects employees’ innovative behavior.

Employees’ innovative behavior consists of the generation, promotion, and application of new ideas, products, processes, or procedures that are intended in the work role, group, or organization (Janssen, 2000). Extant studies have indicated that leadership has a significant impact on employee work-related outcomes (Fu et al., 2020; Islam et al., 2020), and more specifically, it can be a powerful source of employees’ innovative behavior (Pieterse et al., 2009; Hunsaker, 2020). Pieterse et al. (2009) argued that transformational leadership is effective in engendering innovative behavior, and Hunsaker (2020) revealed that spiritual leadership positively predicts innovative behavior. Considering that the majority of existing studies have examined the effects of traditional leadership styles that focus on interactions with subordinates, we extend the extant research by exploring whether responsible leadership that responds to the claims of broader stakeholders can foster employees’ innovative behavior. Since leadership grounded in morality and social responsibility can be a potential predictor of innovation (Tu and Lu, 2013), virtue-oriented responsible leadership may be an important antecedent of employee innovative behavior.

Furthermore, this study explores the underlying mechanisms through which responsible leadership fuels innovative behavior. Scholars have recognized the critical role of leaders in shaping employees’ perceptions of the intended HR practices and facilitating desirable outcomes (Bowen and Ostroff, 2004; Nishii and Paluch, 2018). Specifically, responsible leadership shares common values (e.g., concern for the environment and communities) with socially responsible human resource management (SRHRM) including a set of HR practices targeting CSR implementation and stakeholders’ welfare improvement (Shen and Zhu, 2011). In other words, responsible leaders may serve as SRHRM implementers and in turn foster more motivated and productive employees (Leroy et al., 2018), which is an unexplored topic that may bridge responsible leadership and SRHRM research. Therefore, it is worthwhile and necessary to examine how responsible leaders affect innovative behavior by implementing SRHRM practices. Additionally, organizational pride associated with the organization’s external reputation has been regarded as an essential strategic asset (Katzenbach, 2003), but how it originates from organization’s active engagement in CSR initiatives and then promote employee’s discretionary behavior such as innovative behavior deserves more attention.

In sum, to address the research problem of whether and how responsible leadership affects employees’ innovative behavior, this study empirically examines this relationship and reveals the underlying mechanisms. Our study contributes to both the theoretical and practical fields in several ways. First, the study verifies the role of responsible leadership in fueling innovative behavior and further elucidates the psychological process by demonstrating the mediating roles of SRHRM and organizational pride. This highlights the effectiveness of responsible leadership in the workplace and extends the limited research on its employee outcomes and psychological mechanisms (Doh and Quigley, 2014; Haque et al., 2019b). Second, this study reveals the positive effects of responsible leadership on strengthening employees’ SRHRM perceptions, thus responding to the call of Leroy et al. (2018) for investigating the impacts of leadership on HRM implementation. Third, we advance the existing literature on SRHRM by introducing responsible leadership as an important antecedent and expanding its outcomes to pride and innovation. From a practical perspective, our study suggests that advocating responsible management practices is conducive to stimulating innovative behavior, especially in Chinese manufacturing firms.

## Theory and Hypotheses Development

Social identity theory posits that people are inclined to categorize themselves and others into social groups and establish a positive self-concept by identifying with groups that enhance their self-esteem (Tajfel and Turner, 1979). Given the centrality of social-relational processes in the notion of responsible leadership, social identity theory is useful to interpret how it works (Haque et al., 2019b). Furthermore, individuals tend to bolster their self-image by identifying with organizations recognized for their social engagement and responsibility (Gond et al., 2010), which subsequently motivates employees to strive for organizational objectives. Therefore, social identity theory is appropriate here to explain how responsible leadership and SRHRM affects employee outcomes. Additionally, social exchange theory posits that individuals’ voluntary actions are motivated by the returns they expect from others (Blau, 1964), explaining the social and psychological process underlying the relationship between employees and their organizations (Shen et al., 2018). Based on the norm of reciprocity, it suggests that the investments and inducements that organizations provide for employees through HR practices inspire employees to reciprocate by engaging in extra-role work behaviors that directly benefit the organization (Gould-Williams, 2007). Thus, we further interpret the SRHRM-innovative behavior link applying the social exchange theory.

### Responsible Leadership and Innovative Behavior

Following Maak and Pless (2006), we define responsible leadership as a values-based leadership that integrates effectiveness objectives with social responsibilities and cultivates a sustainable relationship with stakeholders inside and outside the organization to achieve mutual benefits. Specifically, responsible leaders act as experts fulfilling organizational performance goals, citizens meeting moral obligations to society, as well as facilitators caring for the needs of employees (Voegtlin et al., 2020). Responsible leadership can be viewed as a distinguishing characteristic that makes an organization appear superior to others, which, according to social identity theory, may generate positive employee outcomes (Gond et al., 2010).

Firstly, responsible leadership pays special attention to social and environmental goals in order to pursue sustainable value creation (Miska and Mendenhall, 2018), which positively affects corporate reputation (Javed et al., 2020). Thus, employees who more strongly identify with their responsible leaders and moral organizations may experience more work meaningfulness and positive affect, which increases the probability of engaging in creative activities (Tu and Lu, 2013; Rego et al., 2014). Secondly, responsible leaders safeguard individual voices, create an inclusive working environment, and empower employees to share their resources and knowledge (Maak and Pless, 2006). In this way, organizations can receive positive feedback on their fair treatment of employees from people outside the firm (Gond et al., 2010), which may enhance employees’ self-esteem and subsequently motivate them to be more willing to exert creative endeavors (Niu et al., 2018). Thirdly, as attractive role models, responsible leaders may affect employees’ work-related motivation more than other leaders (Haque et al., 2019b), thus stimulating innovative behavior in the workplace. Based on the above, we posit that responsible leadership fosters innovative behavior.

*Hypothesis 1.* Responsible leadership is positively related to innovative behavior.

### The Mediating Role of Socially Responsible Human Resource Management

SRHRM contains three components: legal compliance HRM that meets the standards of labor law (e.g., working hours), employee-oriented HRM that provides employees with organizational support (e.g., career development), and general CSR facilitation HRM that helps companies to engage in external CSR activities (e.g., environmental protection) (Shen and Zhu, 2011). This study focuses on employee-perceived SRHRM because the effectiveness of HRM can not be ascertained unless experienced positively by employees (Wright and Nishii, 2006). Moreover, leaders are widely recognized as critical implementers of HR practices who can shape employees’ perceptions of HRM (Nishii and Paluch, 2018). For example, Ahmad et al. (2021) found that ethical leadership could reinforce the adoption of green HRM (GHRM) because they have common origins in ethics. In a responsible leadership situation, leaders are more likely to provide resources and support for implementing SRHRM rather than other HR systems that only aim to improve employee performance. Hence, responsible leaders may contribute to articulating and conveying the intended messages of SRHRM through daily interactions with employees. Similarly, Ur Rehman et al. (2021) suggested that responsible leadership may promote environmental management practices. Therefore, we consider that responsible leadership can strengthen employees’ perceptions of SRHRM.

SRHRM encourages employees to engage in more external CSR activities, signing to employees that their organization adheres to moral values (Abdelmotaleb and Saha, 2020). Drawing on social identity theory, employees who identify with the organization conforming to social norms and valuing the external reputation may be more willing to exhibit extra-role work behaviors such as innovative behavior (Niu et al., 2018). In addition, based on social exchange theory, the employee-oriented practices of SRHRM facilitate the welfare and meets the concerns of employees, thus leading employees to reciprocate by engaging in more extra-role work behaviors that benefit organizations (Newman et al., 2015). Specifically, SRHRM emphasizes fair working conditions, employee involvement, and communication openness (Shen and Zhu, 2011), which enhances employees’ perceived organizational support and stimulates individuals’ trust in the organization (Jia et al., 2019). In doing so, employees may be more likely to feel obliged to reciprocate for what their organizations have provided by increasing their creative endeavors to improve their work. Based on the above, we hypothesize that perceived SRHRM plays a mediating role between responsible leadership and innovative behavior.

*Hypothesis 2a.* Responsible leadership is positively related to perceived SRHRM.

*Hypothesis 2b.* Perceived SRHRM mediates the positive relationship between responsible leadership and innovative behavior.

### The Mediating Role of Organizational Pride

According to social identity theory, pride mainly originates from the distinctiveness and prestige of the groups that individuals belong to (Tajfel and Turner, 1979). Organizational pride comprises feelings of admiration, importance, and value based on the status evaluations made by employees (Todd and Kent, 2009). Since responsible leadership is usually positively associated with a higher external reputation (Javed et al., 2020), it can be a powerful source of organizational pride. Doh et al. (2011) argued that employees’ pride in the organization is likely to decrease if they perceive the absence of responsible leadership. Moreover, Mousa (2017) found that responsible leadership can make employees feel proud of continuing their membership in their organization. Accordingly, we hypothesize that responsible leadership improves employees’ organizational pride.

Furthermore, employees’ self-respect and positive emotions from their organizational membership will broaden their thought processes and further increase the possibility of generating creative ideas (Fredrickson, 2001). Scholars have also recognized that organizational pride may have the potential to enhance employee creativity (Gouthier and Rhein, 2011; Durrah et al., 2020). Based on the above, employees who are managed by responsible leaders will develop more organizational pride and, therefore, are more likely to undertake innovative activities. Hence, we assume that organizational pride plays a mediating role.

*Hypothesis 3a.* Responsible leadership is positively related to organizational pride.

*Hypothesis 3b.* Organizational pride mediates the positive relationship between responsible leadership and innovative behavior.

### The Sequential Mediation Mechanism

As explained in our justification for hypothesis 2a, responsible leadership may be a promoter of SRHRM. Furthermore, resources gained from SRHRM can translate to pride in organizations (Luu, 2021), which subsequently motivates employees to exhibit more innovative behavior as we discussed in hypothesis 3b. To integrate the hypotheses introduced above, we further posit the sequential mediating effects of SRHRM and organizational pride. Specifically, responsible leadership may increase and reinforce the benefits of SRHRM by strengthening employees’ positive CSR perceptions, which may ultimately boost employees’ pride in membership and engender more innovative efforts.

*Hypothesis 4.* Perceived SRHRM and organizational pride sequentially mediate the positive relationship between responsible leadership and innovative behavior.

**FIGURE 1:**
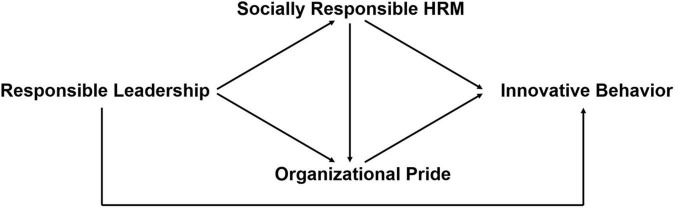
Theoretical model.

Taken together, our hypothesized theoretical model is presented in Figure 1.

## Materials and Methods

### Sample and Procedure

The rapid industrial modernization and economic reform have resulted in both pressure and drivers for Chinese firms to balance economic, environmental, and social performance by addressing CSR issues (Zhu and Sarkis, 2004). Since the manufacturing industry is usually viewed to have the most direct and observable impact on CSR (Liao and Zhang, 2020), a study of socially responsible management (e.g., responsible leadership and SRHRM) may help firms in the manufacturing industry to improve sustainable development (Wang et al., 2021). In this context, we gathered data from manufacturing firms in China (Guangdong province, Jiangsu province, and Shandong province, etc.). Our participants included full-time frontline employees in non-management positions consistent with previous studies (Han et al., 2019; Haque et al., 2019b; Ullah et al., 2021). We conducted a power analysis (effect size of 0.15 and error probability of 0.05), and a size of 200 was deemed sufficient, which is also in line with the sample size required to test a model with four variables (Hair et al., 2009). To obtain a sample that is representative of the population, a total of 400 questionnaires were randomly distributed online. Before the formal investigation, we apprised all participants that our procedure conformed to ethical standards and every respondent would receive a reward of 10 yuan after his or her questionnaire was finally accepted. The investigation lasted from September 2020 to November 2020.

Multiple precautionary measures were taken to minimize common method variance (Podsakoff et al., 2003). Firstly, we ensured that all respondents were participating in the survey voluntarily and demonstrated the research intention and confidentiality statement. Secondly, we reordered the scales and included some attention screening questions in the questionnaire. The data were collected in two separate stages and the time lag was 2 months. To accurately match the completed questionnaires in two phases, every respondent was assigned a unique ID within which the digits were different from each other. We first administered 400 questionnaires on responsible leadership, SRHRM, and demographic information. In total, 366 responses were received, yielding a response rate of 91.5%. Two months later, we distributed questionnaires on organizational pride and innovative behavior to the 366 initial respondents. In total, 304 responses were received, implying a response rate of 83.1%. After matching, we eliminated the invalid questionnaires (those completed in less than 120 seconds or failed to pass the screening questions) and eventually obtained 280 valid responses, with a useful response rate of 70.0%.

Overall, among the 280 participants, 56.4% were male and 43.6% were female; the average age was about 31 years (*SD* = 5.36); the average tenure in their company was 7.47 years (*SD* = 7.17), and 72.5% of the respondents had at least a bachelor’s degree.

### Measures

The scales we chose were originally developed in English and empirically validated in previous research. To ensure meaning accuracy, we translated the English items into Chinese through a back-translation process following the cross-cultural translation procedure (Brislin, 1986). After a pilot study, we made minor revisions to the item wording to increase the content validity. All measures were rated on a five-point Likert scale ranging from 1 (strongly disagree) to 5 (strongly agree).

#### Responsible Leadership

We assessed responsible leadership using Voegtlin (2011) five-item scale developed from the relationship perspective. A sample item is “My direct supervisor demonstrates awareness of the relevant stakeholder claims”. The Cronbach’s α in this study was 0.81.

#### Socially Responsible Human Resource Management

SRHRM was measured by Shen and Benson (2016) six-item scale, which has high reliability and validity in the Chinese context. A sample item is “My company considers candidates’ general attitudes toward CSR in selection”. The Cronbach’s α in this study was 0.88.

#### Organizational Pride

We adopted Gouthier and Rhein (2011) three-item scale of attitudinal organizational pride based on an extensive literature review, qualitative research, and exploratory efforts. A sample item is “I feel proud to work for my company”. The Cronbach’s α in this study was 0.76.

#### Innovative Behavior

The six-item scale developed by Scott and Bruce (1994) was applied to measure innovative behavior. A sample item is “I generate creative ideas”. The Cronbach’s α in this study was 0.87. The self-reported scale of innovative behavior was adopted for several reasons. Firstly, the assessment of innovative behavior as discretionary work behavior is much like a subjective performance appraisal that may vary across different raters, and the supervisor measurement may miss many genuine employee innovative activities and capture only those impressing the supervisors (Organ and Konovsky, 1989). Secondly, the employees’ cognitive reports of their innovative behavior may be more subtle because employees have much more information about the historical, contextual, intentional, and other backgrounds of their own work activities (Jones and Nisbett, 1987). Thirdly, the high reliability of the self-reported scale of Scott and Bruce (1994) has been demonstrated in the extant research (Janssen, 2000; Tu and Lu, 2013; Zhang and Yang, 2020). Then, in line with these studies, the self-reported scale of innovative behavior was applied in our study.

#### Control Variables

Following previous research (Janssen, 2000; Tu and Lu, 2013; Niu et al., 2018; Hunsaker, 2020; Zhang and Su, 2020; Zhang and Yang, 2020), we incorporated employee gender, age, education, and tenure in the company as control variables when testing the hypotheses.

## Results

### Reliability and Validity Analyses

Several factor analyses were conducted to test the reliability and validity of our measurement model (Table 1). First, the Kaiser-Meyer-Olkin (KMO) value (>0.7) and the significant results of Bartlett’s test indicate that it is plausible for us to perform factor analyses. Second, factor loadings of all variables exceed the recommended value of 0.5 with the total variance explained of all variables surpassing 50% (Hair et al., 2010). Third, considering that the Cronbach’s α (>0.7) and the composite reliability (CR) (>0.7) ensure the internal consistency of our measures, the average variance extracted (AVE) of study variables (>0.4) suggests that the convergent validity is also acceptable (Fornell and Larcker, 1981).

**TABLE 1:** Results of CRs, AVEs, Cronbach’s α, and total variance explained.

	KMO	Bartlett’s test	Loadings	Total variance explained	Cronbach’s α	CR	AVE
RL	0.82	416.40 (10)***	0.64–0.72	57.11%	0.81	0.81	0.47
SRHRM	0.89	855.20 (15)***	0.60–0.83	63.48%	0.88	0.89	0.57
OP	0.70	215.14 (3)***	0.67–0.78	68.21%	0.76	0.77	0.53
IB	0.88	735.77 (15)***	0.70–0.75	61.09%	0.87	0.87	0.53

*N = 280.*

*RL, responsible leadership; SRHRM, socially responsible HRM; OP, organizational pride; IB, innovative behavior; KMO, Kaiser-Meyer-Olkin; CR, composite reliability; AVE, average of variance extracted.*

***p < 0.001.

Furthermore, the results of a series of confirmatory factor analyses (CFA) confirm the discriminant validity of our measurement model (Table 2). Specifically, the hypothesized four-factor model significantly performs better than the three-factor, two-factor, and one-factor models. These results reveal that our study variables are distinguishable. Moreover, the square roots of the AVE for all variables exceed the correlations between the focal variable and other variables (Table 3), further indicating adequate discriminant validity (Fornell and Larcker, 1981). Altogether, our measurement model demonstrates satisfactory reliability and validity.

**TABLE 2:** Results of confirmatory factor analyses.

Model	χ^2^	df	χ^2^/df	RMSEA	SRMR	CFI	TLI
5-Factor model (RL; SRHRM; OP; IB; CMV)	270.804	145	1.868	0.056	0.040	0.955	0.941
4-Factor model (RL; SRHRM; OP; IB)	336.320	164	2.051	0.061	0.048	0.938	0.928
3-Factor model (RL + SRHRM; OP; IB)	487.139	167	2.917	0.083	0.056	0.885	0.869
2-Factor model (RL + SRHRM; OP + IB)	591.266	169	3.499	0.094	0.065	0.848	0.829
1-Factor model (RL + SRHRM + OP + IB)	739.469	170	4.350	0.069	0.109	0.795	0.771

*N = 280.*

*RL, responsible leadership; SRHRM, socially responsible HRM; OP, organizational pride; IB, innovative behavior; CMV, common method variance; df, degrees of freedom; RMSEA, root mean square error of approximation; SRMR, standardized root mean square residual; CFI, comparative fit index; TLI, Tucker-Lewis index.*

**TABLE 3:** Means, standard deviations, correlations, and AVE square root values.

Variables	*M*	*SD*	1	2	3	4	5	6	7	8
1. Gender^a^	1.436	0.497	–							
2. Age	31.770	5.358	−0.062	–						
3. Education^b^	2.714	0.653	−0.024	−0.124*	–					
4. Tenure	7.471	7.173	−0.055	0.508**	−0.071	–				
5. RL	4.019	0.538	−0.115	−0.077	0.073	−0.001	**0.682**			
6. SRHRM	4.126	0.621	−0.130*	−0.048	−0.027	0.054	0.619**	**0.756**		
7. OP	4.332	0.558	−0.093	0.057	−0.001	0.129*	0.621**	0.535**	**0.726**	
8. IB	4.018	0.602	−0.144*	−0.040	0.106	0.067	0.635**	0.572**	0.566**	**0.729**

*N = 280. Bold numbers on the diagonal line are the square root values of the AVE for each variable.*

*RL, responsible leadership; SRHRM, socially responsible HRM; OP, organizational pride; IB, innovative behavior.*

*^a^1 = male, 2 = female.*

*^b^1 = high school and below, 2 = junior college, 3 = undergraduate, 4 = postgraduate.*

*p < 0.05; **p < 0.01.

### Common Method Variance Examinations

Several statistical methods were adopted to examine the potential common method variance in our study. Firstly, Harmon’s one-factor test was conducted with exploratory factor analyses (EFA) and CFA respectively (Podsakoff et al., 2003). Applying the unrotated solution, the results indicate that the first factor makes up 43% of the explained variance (<50%), which reveals that there is no single factor playing a major role in interpreting the variance of the dependent variable (Podsakoff et al., 2012). Meanwhile, according to the results of CFA (Table 2), the one-factor model exhibits the worst performance compared to others. These results initially verify that our data is not biased by the common method variance.

Furthermore, we adopted the unmeasured latent method construct (ULMC) technique (Podsakoff et al., 2003) that has been widely used to test the common method variance in previous research (Bozionelos and Simmering, 2021). Specifically, when conducting the CFA, we further added an extra latent variable named ‘CMV’ on which all items of the four theoretical constructs were loaded (Table 2). Compared with the hypothesized four-factor model, the TLI indices of the five-factor model with CMV only increased by 0.01, which is below the recommended cut-off point of 0.05 (Bagozzi and Yi, 1990; Bozionelos and Simmering, 2021). The results above further demonstrate that the effects of CMV on estimates are not significant.

In sum, the common method variance is not an obvious problem in the present study and does not invalidate our research findings.

### Descriptive Statistics and Correlations

Applying IBM SPSS Statistics 21.0, we obtained the results of means, SDs, and correlations of all variables (Table 3). The moderately high correlations between variables provide preliminary support for our hypotheses. Specifically, responsible leadership shows a significant positive correlation to SRHRM (*r* = 0.619, *p* < 0.01), organizational pride (*r* = 0.621, *p* < 0.01), and innovative behavior (*r* = 0.635, *p* < 0.01). SRHRM is positively correlated with organizational pride (*r* = 0.535, *p* < 0.01) and innovative behavior (*r* = 0.572, *p* < 0.01). Organizational pride is positively related to innovative behavior (*r* = 0.566, *p* < 0.01).

### Hypotheses Testing

We tested the hypotheses by employing hierarchical regression analyses and PROCESS macro that is widely used by scholars to examine the general and sequential mediation through the bootstrap method (Hayes, 2013).

Hypothesis 1 assumes the main effects of responsible leadership on innovative behavior. As shown in Table 4, with demographic variables controlled, responsible leadership has a significant positive effect on innovative behavior (Model 6: β = 0.622, *p* < 0.001). Thus, hypothesis 1 is supported.

**TABLE 4:** Results of hierarchical regression analyses.

		SRHRM		Organizational pride		Innovative behavior			
		Model 1	Model 2	Model 3	Model 4	Model 5	Model 6	Model 7	Model 8
**Control variables**									
	Gender	−0.166	−0.076	−0.097	−0.026	−0.171*	−0.092	−0.059	−0.082
	Education	−0.035	−0.071	0.004	−0.024	0.091	0.060	0.091*	0.069
	Age	−0.013	−0.006	−0.002	0.004	−0.011	−0.004	−0.002	−0.006
	Tenure	0.009	0.006	0.010	0.008	0.010	0.007	0.005	0.004
**Independent variables**									
	RL		0.709***		0.558***		0.622***	0.308***	0.404***
**Mediators**									
	SRHRM							0.444***	
	OP								0.392***
	F	2.062	36.016***	1.706	24.123***	3.026**	28.651***	40.331***	35.038***
	R^2^	0.029	0.397	0.024	0.306	0.042	0.343	0.470	0.435
	ΔR^2^	0.029	0.368***	0.024	0.282***	0.042**	0.301***	0127***	0.092***

*N = 280. Coefficients are unstandardized.*

*RL, responsible leadership; SRHRM, socially responsible HRM; OP, organizational pride.*

*p < 0.05; **p < 0.01; ***p < 0.001.

Hypotheses 2 and 3 predict the mediating effects of SRHRM and organizational pride, respectively. As displayed in Table 4, responsible leadership is positively related to SRHRM (Model 2: β = 0.709, *p* < 0.001) and organizational pride (Model 4: β = 0.558, *p* < 0.001), thus confirming hypotheses 2a and 3a. Responsible leadership remains positively related to innovative behavior when adding mediators SRHRM (model 7: β = 0.308, *p* < 0.001) and organizational pride (model 8: β = 0.404, *p* < 0.001). Accordingly, the mediating roles of SRHRM and organizational pride are initially supported.

Furthermore, given that the bootstrap method can rule out the shortage of ordinal regression when examining the significance of a mediating path (Fritz and MacKinnon, 2007), we further applied PROCESS Model 6 with 10,000 bootstrap samples and bias-corrected 95% confidence interval (CI) to test the mediating effects of SRHRM and organizational pride. As demonstrated in Table 5, for the first path, i.e., mediation through SRHRM only, the 95% CI is [0.149,0.362], excluding 0. Likewise, for the second path, i.e., mediation through organizational pride only, the 95% CI is [0.020,0.112], not containing 0. Therefore, hypotheses 2b and 3b are verified.

**TABLE 5:** Results of mediation test using bootstrap.

		Effect	SE	LL 95% CI	UL 95% CI
**Outcome: IB**					
	Constant	1.443	0.223	1.004	1.883
	RL	0.641	0.055	0.532	0.749
**Outcome: SRHRM**					
	Constant	1.250	0.221	0.816	1.684
	RL	0.716	0.054	0.609	0.823
**Outcome: OP**					
	Constant	1.571	0.203	1.171	1.971
	RL	0.253	0.604	0.134	0.372
	SRHRM	0.423	0.052	0.320	0.526
**Outcome: IB**					
	Constant	0.517	0.229	0.067	0.967
	RL	0.264	0.064	0.139	0.389
	SRHRM	0.341	0.059	0.224	0.458
	OP	0.238	0.613	0.118	0.359
**Indirect effects**					
	RL→SRHRM→IB	0.244	0.055	0.149	0.362
	RL→OP→IB	0.060	0.024	0.020	0.112
	RL→SRHRM→OP→IB	0.072	0.021	0.032	0.115

*N = 280.*

*RL, responsible leadership; SRHRM, socially responsible HRM; OP, organizational pride; IB, innovative behavior. LL, lower limit; UL, upper limit; CI, confident interval.*

Hypothesis 4 posits that SRHRM and organizational pride sequentially mediate the responsible leadership-innovative behavior relationship. The 95% CI for the sequential mediating path is [0.032,0.115] with 0 outside (Table 5). Thus, hypothesis 4 is supported.

Overall, the obtained results conform to all the proposed hypotheses.

## Discussion

This paper underlines the significance of responsible leadership and further reveals the psychological mechanisms through which it fuels innovative behavior. Consistent with the hypotheses, we find that responsible leadership exerts a direct positive influence on innovative behavior, perceived SRHRM, and organizational pride. Moreover, perceived SRHRM and organizational pride respectively and sequentially transmit the impacts of responsible leadership on innovative behavior. Our findings may provide several theoretical and managerial implications.

### Theoretical Implications

This study enriches and develops the existing literature in the following ways. First, we empirically extend the current literature on responsible leadership by providing direct evidence for its effectiveness and revealing the psychological mechanisms through which it functions. Specifically, given few studies have examined the employee work outcomes of responsible leadership in China where responsibility and innovation are especially emphasized (Walumbwa et al., 2011), our study verifies the positive effects of responsible leadership on employee innovative behavior in the Chinese manufacturing industry, responding to the call for responsible leadership research in China (Huang et al., 2020). This finding indicates the generalizability and external validity of responsible leadership and innovative behavior that is originally developed and mainly studied in the western context. Additionally, by showing what leaders can achieve through responsible behaviors, this study enhances our understanding of the power of responsible leadership (Tsui, 2019) and may also strengthen confidence in the positive models of leadership (Voegtlin et al., 2020). Furthermore, we unfold the responsible leadership-innovative behavior relationship by testing two mediators (perceived SRHRM and organizational pride) from both cognitive and emotional perspectives, which enriches the scarce research on how responsible leadership works at the individual level (Haque et al., 2019a). Altogether, our findings develop the existing knowledge on how and why responsible leaders can play a crucial role in the workplace and inspire further research on alternative mechanisms.

Second, we advance the burgeoning research on how leaders are involved in shaping the effectiveness of HRM practices. Although integrating leadership theory with HRM research is regarded as a prime area for future inquiry, a surprising dearth of studies has explored the leadership-HRM relationship (Steffensen et al., 2019). Unlike Gond et al. (2011) who explored the contribution of HRM to responsible leadership, we concentrate on leaders’ subjective initiative and verify that responsible leadership could be an effective promoter of SRHRM. This lends empirical support to the synergistic perspective that managerial leadership may influence the availability and reinforcement of HRM practices (Steffensen et al., 2019). In addition, our finding that responsible leadership strengthens employees’ perceptions of intended SRHRM information also develops the argument that leader behaviors can help bridge the gap between intended HRM and perceived HRM (Nishii and Paluch, 2018). Furthermore, this study indicates that leaders’ values and attitudes can affect their adoption and implementation of HRM content, encouraging researchers to explore different roles of other leadership styles in HRM implementation.

Third, this study simultaneously broadens the limited research on antecedents and outcomes of SRHRM. Although SRHRM is essential for organizational sustainability, it has received insufficient attention yet (Shen and Zhang, 2019). In response to the call for investigating the formation mechanism of SRHRM such as leaders’ CSR attitude (Zhao et al., 2021), we identify a new predictor of SRHRM by revealing the role of responsible leadership, which develops our understanding of SRHRM implementation and encourages more scholarly attention on motivators of SRHRM from leadership perspectives. Additionally, regarding outcomes, different from the prior studies focusing on pro-social outcomes such as support for external CSR (Shen and Zhang, 2019), our study indicates that SRHRM can boost pride and innovation. In short, our findings not only make up for the deficiency of research on SRHRM but also reveal the benefits of socially responsible management practices.

### Practical Implications

In addition to implications for theory, this study also provides important practical implications for firms and managers, especially in the Chinese manufacturing industry. First, our findings indicate that responsible leadership can be an efficient catalyst for employees’ innovative behavior. Hence, firms should recruit and cultivate more responsible leaders, conduct training programs to improve leaders’ skills in stakeholder communication, and provide responsible leaders with better promotion opportunities (Agarwal and Bhal, 2020). Meanwhile, establishing a stakeholder culture or promoting ethical values in organizations may also help to shape the organizational context for the exercise of responsible leadership. Furthermore, our findings indicate that supervisors’ responsible behaviors can be efficient motivators of positive employee work outcomes. Therefore, leaders at different levels should be aware of their responsibilities to all internal and external stakeholders, thus facilitating employees’ organizational pride and spirit of innovation.

Second, implementing and advocating SRHRM pays off. With the goal of effective SRHRM implementation, we advise firms to provide incentives such as linking employees’ social performance to performance appraisals or rewards to motivate greater support and involvement in external CSR activities. As such, organizations can improve their reputation and enhance employees’ pride in organizations. Furthermore, organizations ought to adopt more employee-oriented practices to promote employee well-being and perceived organizational support. For instance, we encourage firms to enhance the staff’s work-life balance through flexible working hours or employment programs. In this way, employees may be inspired to exert more innovative efforts to achieve organizational objectives.

Third, CSR strategies can be communicated effectively within organizations through responsible leadership and SRHRM. Managers ought to integrate CSR issues in their leadership skills by considering the claims of various stakeholders in the decision-making process and act as role models for employees through socially responsible behaviors. Additionally, organizations should pay more attention to incorporating CSR values into HR policies and practices, thus stimulating employee organizational pride and support for external CSR policies. The alignment of responsible leadership with SRHRM is important in that it helps to strengthen individual CSR perceptions and fuel innovation in organizations.

### Limitations and Future Directions

First, because we focus on individual perceptions to explore underlying psychological mechanisms, the scales applied in our research were reported by employees. Hence, to minimize the possible common method bias, multi-source data could be considered in future research. Second, we encourage future researchers to validate our findings in multiple industrial or cultural backgrounds and consider conducting longitudinal surveys or experiments to help justify the cause-and-effect relationship. Third, our theoretical model can be further improved by the inclusion of other mediators (trust in leader, harmonious work passion, felt obligation for constructive change, etc.), which may provide diversified underlying mechanisms interpreting how responsible leadership affects innovative behavior. Fourth, we expect future research to include moderators such as organizational culture or individual characteristics to advance our knowledge on the contingent effectiveness of responsible leadership. For example, testing whether the stakeholder culture in organizations can attenuate or strengthen the positive effects of responsible leadership on SRHRM would add value to the present study. Finally, given the multilevel nature of responsible leadership (Miska and Mendenhall, 2018), more in-depth studies on how responsible leadership affects outcomes at higher levels or across levels (e.g., team and organizational performance) are also warranted.

## Conclusion

Based on social identity theory and social exchange theory, this study sought to explore whether and how responsible leadership fuels employees’ innovative behavior. The analytic results confirm the positive relationship between responsible leadership and innovative behavior. Moreover, the independent and sequential mediating roles of SRHRM and organizational pride are further captured. Our findings lend direct support to the effectiveness of responsible management practices, indicating the external validity of responsible leadership and innovative behavior in the Chinese context. Furthermore, this study provides insights into its underlying mechanism, bridging the gaps between responsible leadership and SRHRM. In practical dimension, our study inspires organizations and managers to promote responsible management practices to achieve long-term innovation and sustainable success.

## Data Availability Statement

The datasets generated for this study are available on request to the corresponding author.

## Ethics Statement

Ethical review and approval was not required for the study on human participants in accordance with the local legislation and institutional requirements. The patients/participants provided their written informed consent to participate in this study.

## Author Contributions

WD conceived the project, performed the data collection and analyses, and wrote the first draft of the manuscript. LZ directed the research and made critical revisions. Both authors approved the final version of the manuscript.

## Conflict of Interest

The authors declare that the research was conducted in the absence of any commercial or financial relationships that could be construed as a potential conflict of interest.

## Publisher’s Note

All claims expressed in this article are solely those of the authors and do not necessarily represent those of their affiliated organizations, or those of the publisher, the editors and the reviewers. Any product that may be evaluated in this article, or claim that may be made by its manufacturer, is not guaranteed or endorsed by the publisher.
